# Effects of low pH on the coral reef cryptic invertebrate communities near CO_2_ vents in Papua New Guinea

**DOI:** 10.1371/journal.pone.0258725

**Published:** 2021-12-15

**Authors:** Laetitia Plaisance, Kenan Matterson, Katharina Fabricius, Sergei Drovetski, Chris Meyer, Nancy Knowlton

**Affiliations:** 1 National Museum of Natural History, Smithsonian Institution, Washington, D.C., United States of America; 2 Laboratoire Evolution et Diversité Biologique, CNRS/UPS, Toulouse, France; 3 Dipartimento di Scienze Biologiche, Geologiche e Ambientali (BiGeA), Università di Bologna, Ravenna, Italy; 4 Australian Institute of Marine Science, Townsville, Queensland, Australia; 5 US Geological Survey, Eastern Ecological Science Center, Beltsville, MD, United States of America; Universita degli Studi di Genova, ITALY

## Abstract

Small cryptic invertebrates (the cryptofauna) are extremely abundant, ecologically important, and species rich on coral reefs. Ongoing ocean acidification is likely to have both direct effects on the biology of these organisms, as well as indirect effects through cascading impacts on their habitats and trophic relationships. Naturally acidified habitats have been important model systems for studying these complex interactions because entire communities that are adapted to these environmental conditions can be analyzed. However, few studies have examined the cryptofauna because they are difficult to census quantitatively in topographically complex habitats and are challenging to identify. We addressed these challenges by using Autonomous Reef Monitoring Structures (ARMS) for sampling reef-dwelling invertebrates >2 mm in size and by using DNA barcoding for taxonomic identifications. The study took place in Papua New Guinea at two reef localities, each with three sites at varying distances from carbon dioxide seeps, thereby sampling across a natural gradient in acidification. We observed sharp overall declines in both the abundance (34–56%) and diversity (42–45%) of organisms in ARMS under the lowest pH conditions sampled (7.64–7.75). However, the overall abundance of gastropods increased slightly in lower pH conditions, and crustacean and gastropod families exhibited varying patterns. There was also variability in response between the two localities, despite their close proximity, as one control pH site displayed unusually low diversity and abundances for all invertebrate groups. The data illustrate the complexity of responses of the reef fauna to pH conditions, and the role of additional factors that influence the diversity and abundance of cryptic reef invertebrates.

## Introduction

Animal diversity on coral reefs is dominated by cryptic organisms that occupy small cavities within the coral limestone [1–3]. This cryptofauna encompasses most metazoan phyla [4] and comprises a large portion of living reef biomass [5]. Although often ignored in ecological studies, the cryptofauna performs a broad array of functional roles, making it vital to the trophic ecology of coral reef ecosystems [3,5,6]. Examples of these roles include the recycling of inorganic nutrients [7] and organic detritus [8], the reduction of algal proliferation *via* herbivory [9], and the nutritional support of planktivores that feed on cryptofaunal larvae [3].

Understanding the response of coral reef cryptofauna to climate change stressors, including the effects of ocean acidification (OA), is thus of critical importance. Many reef organisms exhibit direct physiological responses to reduced pH [10,11], but cryptofauna could be particularly sensitive to the indirect effects of ocean acidification because of their dependence on the small interstices amongst the reef framework for shelter. Studies of naturally acidified reef systems have shown reduced reef topographical complexity due to the dominance of structurally simpler species such as boulder corals [12,13] and increased bioerosion rates of dead coral skeletons and the reef matrix [14,15]. Other studies have demonstrated shifts from calcifying bioconstructors to non habitat-forming communities [16,17]. These changes can alter reef-associated assemblages as well as facilitate the proliferation of more opportunistic species [18]. Thus coral reefs naturally exposed to elevated levels of carbon dioxide (CO_2_) provide an important opportunity to test whether ocean acidification and related changes in water chemistry influences reef cryptofauna, directly through physiological responses to the altered seawater carbonate chemistry as well as indirectly through effects such as reduced habitat complexity [19,20].

The volcanic CO_2_ seeps in Milne Bay, Papua New Guinea (PNG) are particularly informative in this regard [12]. Located in the heart of the Coral Triangle, these seeps have been well described by past studies. Notably, they are characterized by an absence of other stressors (*e*.*g*. high seawater temperature, concentrations of heavy metals or methane) that could interfere with the ability to tease apart the impacts of changes in seawater carbonate chemistry and associated pH changes [12,21–24]. These studies demonstrated the similar physico-chemical conditions between seep and adjacent control sites. These characterictics explain why the PNG CO_2_ seeps are therefore an important field site to characterize the OA impacts on various reef associated taxa and ecological processes.

However, few studies have investigated the invertebrate cryptofauna at the PNG seeps or other naturally acidified coral reef ecosystems. In PNG, recent studies have focused on large benthic invertebrates [12], demersal plankton [25], and invertebrate recruitment success [26]; these studies reported decreases in the abundance and diversity of species and higher taxonomic groups (families up to phyla). Based on these studies, it can be expected that cryptofaunal invertebrate communities will also be impacted by changing seawater carbonate chemistry with implications on abundance, diversity and community composition. Given the array of functional roles and the importance of cryptic invertebrate communities to the trophic ecology of tropical reefs, the paucity of empirical data on the composition of cryptofaunal assemblages under low pH conditions represents a major gap in our understanding of how coral reef communities will change with OA [27].

**Fig 1:**
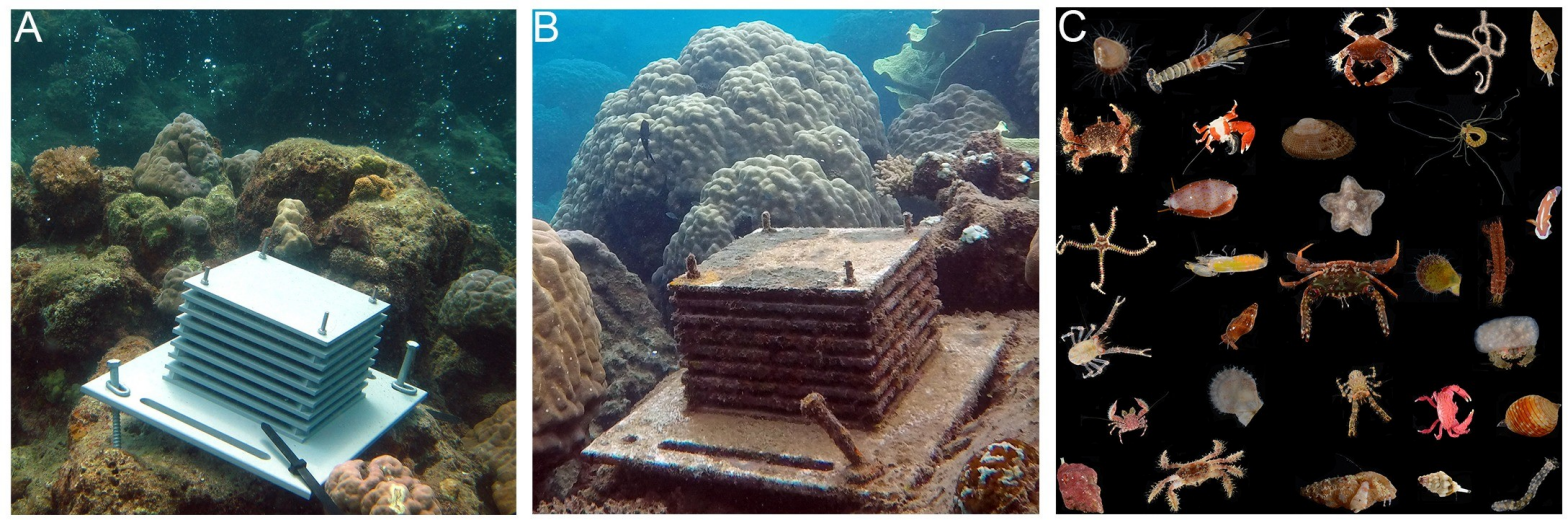
ARMS invertebrate sampling. Sampling in Upa Upasina showing A) ARMS at the time of deployment in low pH, B) ARMS after two years of deployment in medium pH, and C) composite photo of a subsample of invertebrate species from one ARMS in control (high) pH.

The lack of information on cryptofauna stems primarily from challenges associated with sampling and identifying these small and highly diverse organisms. To address these challenges, we used methods developed for previous reef cryptofaunal studies that coupled standardized sampling using Autonomous Reef Monitoring Structures (ARMS; Fig 1) [28,29] with DNA-based identification. We studied the diversity of the cryptofauna at two localities in Papua New Guinea that each exhibited sites with varying degrees of influence from carbon dioxide emerging from volcanic seeps in the sea floor. At each of the two localities, replicate ARMS were placed at each of three pH sites [control (high), medium, and low pH].

ARMS are artificial, standardized sampling units whose structural complexity promotes recruitment of a diverse array of sessile and mobile organisms (Fig 1C). For this reason, they have been used to investigate cryptofaunal diversity in temperate and tropical environments around the world [28–33]. Using ARMS for sampling has the advantage of being non-destructive, as cryptic organisms do not need to be extracted from the reef matrix, and the standardized approach facilitates comparisons across sites, time and environmental conditions. ARMS are particularly useful in areas where natural substrate (e.g. coral rubble) is rare or where reef structure differs greatly across sites, as is the case in studies comparing reefs growing in typical pH conditions with acidified reefs, where structurally complex corals and coral rubble are rare.

## Materials and methods

### 1. Study site

This study was conducted in Milne Bay Province, Papua New Guinea at two shallow-water localities (Dobu and Upa Upasina) where almost pure (~99%) CO_2_ gas seeps through the seafloor and into the water, changing the water carbonate chemistry and creating sharp gradients in seawater carbonate chemistry parameters within a short distance [12,21–24,26]. We selected the position of ARMS deployments based on the seawater carbonate chemistry maps previously documented by Fabricius *et al*. [21]. We chose two reduced pH levels (low and medium pH) within each of the two seep sites (Dobu, coordinates: S9° 44.20’, E150° 52.06’; Upa Upasina, coordinates: S9° 49.45’, E150° 49.06’). We selected two nearby control (high) pH sites located ~2.5 km away from the seep zone in Dobu (coordinates: S9° 45.13’, E150° 51.22) and ~0.5 km away from the seep zone in Upa Upasina (coordinates: S9° 49.69’, E150° 49.23’). All six sampling sites were very similar in their environmental conditions, including temperature, salinity, light and currents [12,21]. Benthic communities and seascapes differed between the control sites and the seep sites by showing a decrease in taxonomic diversity and a progressive loss of structural complexity owing to the dominance of massive corals (*Porites* sp.) in altered pH conditions, as documented by several previous studies [*e*.*g*. 12,21,26].

To confirm the pH and water chemistry values at each of the ARMS deployment sites, seawater samples were collected during four field expeditions in 2012, 2013 and 2014 and values for total alkalinity and dissolved inorganic carbon were measured for a median of seven samples per ARMS site (n range: 2–17). Additionally, pH dataloggers (Sea-bird Scientific and SeaFET sensors) were deployed over 24-hour periods in the vicinity of the ARMS to measure *in situ* pH and temperature (S1 Table).

### 2. ARMS deployment, sample collection and processing

ARMS units (23 cm x 23 cm x 17 cm) were composed of nine grey PVC plates separated by 12.7 mm spacers forming eight alternating open and obstructed layers (Fig 1A) [28,29,34]. A total of 36 units were deployed at a depth of 4–5 m in April 2012 at three pH conditions (S1 Table) in Dobu (low pH: 7.64, medium pH: 7.85, control pH: 7.99) and in Upa Upasina (low pH: 7.75, medium pH: 7.85, control pH: 8.01). At each pH site, six replicate ARMS were positioned approximately 5 m apart. A total of twelve ARMS were retrieved after 24 months of deployment time (3 ARMS each in control pH and in medium pH at Upa Upasina, and 3 ARMS each in control pH and low pH at Dobu). Eighteen ARMS were retrieved after 30 months of deployment time (3 ARMS in all three pH conditions at both Dobu and Upa Upasina). Six ARMS could not be retrieved because of the limited amount of ship time available during the second of the three expeditions at this remote location.

For retrieval, a 40 μm mesh-lined crate was placed over the ARMS to prevent the loss of motile organisms, and both were transferred to a disassembly bin filled with filtered (40 μm) and aerated seawater. The crate was then removed and the ARMS was carefully disassembled by removing plates individually from the structure and rinsing in the disassembly water. All motile organisms >2 mm were removed from each plate, and the disassembly seawater was then passed through a series of three sieves (2 mm, 500 μm and 106 μm) to collect and separate unattached invertebrates according to body size. The sessile organisms were photographed and scraped from the plates. This study reports on the >2 mm motile organisms; the organisms in the 500 μm and 106 μm sieves and the scrapings were preserved for further analyses and are not presented here. For each ARMS, all organisms >2 mm in size were sorted to morphospecies in the field. Each morphospecies was identified to the lowest taxonomic rank possible (order-level or lower depending on the taxon), photographed, subsampled for genetic analysis, and preserved in 95% ethanol.

To determine the relationship between the ARMS community and the community of invertebrates associated with natural substrates, we also collected a limited number (n = 3) of dead coral heads [*Pocillopora damicornis (Linnaeus*, *1758)*] at the control/high and medium pH sites at Upa Upasina during the time of the initial ARMS deployment; this could not be done at the low pH site in Upasina nor in Dobu due to a scarcity of dead *Pocillopora* heads. For dead coral heads, only crustaceans >2 mm were collected; the methods of collection were as described for previous studies of crustaceans associated with dead coral heads [29,35] and preservation methods were as described for the ARMS samples above.

The collection and exportation permit was granted by the Department of Environment and conservation, Boroko, Papua New Guinea (permit number: 014284).

### 3. Molecular analysis

DNA was extracted from tissue subsamples using a standard proteinase-k digestion followed by phenol-chloroform extraction on an AutoGenprep 965 (Autogen, Holliston, MA, USA). The standard Cytochrome Oxidase Subunit I (COI) barcoding fragment (658 bp) [36,37] was amplified using the jgLCO1490/jgHCO2198 primer pair [38]. PCR reactions were prepared using 5 μl of GoTaq Hot Start Mastermix (Promega, Madison, WS, USA), 0.3 μl of each primer (10μM), and 1 μl of genomic DNA in a final reaction volume of 10 μl. PCR cycling conditions were as follows: 7 min at 95°C; 4 cycles of 30 s at 94°C, 45 s at 50°C, and 60 s at 72°C; 34 cycles of 30 s at 94°C, 45 s at 45°C, and 60 s at 72°C; and a final extension of 8 min at 72°C. PCR products were visualized on 1.5% agarose gels and purified using ExoSAP-IT (Affymetrix, Santa Clara, CA, USA). Automated sequencing was performed in both directions directly on purified PCR products using Applied Biosystems BigDye terminator V3.1 (Thermo Fisher Scientific, Foster City, CA, USA). Sequence reactions were purified using Millipore 96-well plates loaded with Sephadex G-50 and run on an ABI 3130xl genetic analyzer (Applied Biosystems, Thermo Fisher Scientific, Foster City, CA, USA).

Sequences were trimmed using Phred score 20 or more than two ambiguous sites withing a 10-site segment using Sequencher v.5.2.4 (Gene Codes Corporation, Ann Harbor, MI, USA). Consensus sequences were assembled automatically, inspected and edited manually. Finalized consensus sequences were blasted against NCBI GenBank (https://blast.ncbi.nlm.nih.gov/Blast.cgi) to identify and remove bacterial contaminants or sequences showing a mismatch with the field description of the organism (e.g. a snail sequence that had been scored as a worm in the field). Those samples were resequenced. A total of 22 specimens corresponding to 1.5% of the total number of sampled individuals (10 bivalves, 7 gastropods and 5 polychaetes) did not produce a sequence and were removed from the analysis. All collected specimens were accessioned into the collections of the National Museum of Natural History, Smithsonian Institution, Washington DC.

### 4. Data analysis

Consensus sequences were clustered into Operational Taxonomic Units (OTUs) in MOTHUR [39] using the furthest neighbor clustering algorithm with a standard 3% dissimilarity threshold for species discrimination [40]. For taxonomic assignment, the centroid sequence of each OTU was queried against the Genbank and BOLD databases using Blastn. Taxonomic assignments were carried out using a 95% and a 97% sequence similarity threshold to obtain identifications at the order and species levels respectively. Identifications were subsequently verified from photos taken in the field.

Community composition statistical analyses revealed no differences among ARMS from the same locality and pH site but collected at different dates, and so these ARMS were pooled in order to produce the composition summary graphs in R version 3.6.1. Rarefaction curves and sample-based non-parametric biodiversity estimators (Chao I and ACE [41]) were calculated using EstimateS 8.2.0 [42]. The use of non-parametric estimators allows for a better estimation of overall alpha diversity in very biodiverse samples when true diversity is unknown and many species remain to be sampled [43]. In order to avoid underestimating overall diversity in the two sites where only three ARMS were processed (Dobu medium pH and Upa Upasina low pH) versus the remaining four sites where six ARMS were sampled, we also report the diversity values computed in EstimateS for n = 3 ARMS per site (subsampling without replacement, 100 randomizations).

We calculated average species richness (S) and Shannon diversity (H) on unrarified OTU abundances using the ‘vegan’ R package [44], and checked for normality (∞ = 0.05) using the Shapiro-Wilk test and variance heterogeneity using the Levene’s test. We evaluated differences in overall cryptofaunal abundance (i.e., number of recovered sequences), OTU richness, and Shannon diversity using one-way ANOVAs with pH as factor for each locality separately. The effect of pH on the abundance of common taxonomic groups was evaluated using non-parametric Kruskal-Wallace tests.

Analysis of community composition among pH conditions was performed at the OTU and family levels using Bray-Curtis dissimilarity matrices on log-transformed abundance data in PRIMER v7 [45] with the PERMANOVA add-on [46]. We used type III SS PERMANOVA (2-way crossed) with site pH level [low, medium, control (high) pH] and locality (Dobu and Upa Upasina) as fixed factors and 1-way PERMANOVA for each locality dataset separately to investigate the effect of pH only at the locality scale. Principal Coordinate Analysis (PCoA) of Bray-Curtis dissimilarities was used to visualize differences among invertebrate assemblages at the OTU and family levels. To better visualize which invertebrate OTUs and families contributed to differences among pH conditions, Pearson’s correlation vectors were overlaid on the PCoA plot for vector lengths greater than 0.6. To identify which taxa contributed the most to the dissimilarity among pH levels and locality, we used a SIMPER analysis on log-transformed abundance data at the family level.

## Results

### 1. Overall abundance

In total, we barcoded 1424 invertebrates from the 30 ARMS units that were retrieved: 905 in Upa Upasina and 519 in Dobu (Table 1). The highest abundance (75.5 individuals per ARMS) was found at the control pH site of Upa Upasina and the lowest number (26.5) at the low pH site of Dobu (Table 1).

**Table 1:** ARMS sampling and diversity summary.

Locality	Upa Upasina				Dobu			
Site	Control	Medium	Low	Combined	Control	Medium	Low	Combined
**Mean pH**	8.01	7.85	7.75	---	7.99	7.85	7.64	---
**# ARMS retrieved at 24/30 months**	3/3	3/3	0/3	6/9	3/3	0/3	3/3	6/9
**Total number of individuals**	453	300	152	905	179	181	159	519
**Total number of OTUs**	137	107	47	213	69	73	71	181
**# Individuals per ARMS (SD)**	75.5 (29.0)	50.0 (24.8)	50.7 (25.1)	---	29.8 (7.3)	60.3 (9.2)	26.5 (10.9)	---
**# OTUs per ARMS (SD)**	35.0 (10.7)	25.8 (8.2)	20.3 (12.7)	---	16.7 (3.2)	32.0 (2.6)	17.5 (6.3)	---
**Shannon (SD)**	3.12 (0.15)	2.91 (0.25)	2.48 (0.52)	---	2.52 (0.15)	3.21 (0.15)	2.67 (0.35)	---
**ACE (SD); n = 3**	181 (44)	140 (23)	104 (0)	---	114 (29)	172 (0)	81 (23)	---
**Chao I (SD); n = 3**	180 (32)	124 (22)	90 (18)	---	110 (17)	187 (43)	81 (34)	---

Summary table representing number and diversity numbers of the cryptic invertebrates retrieved from the ARMS across the two localities each with three pH levels (high, medium and low pH) and diversity results for the six pH sites. For each pH site, mean abundance, richness and diversity were computed per ARMS with standard deviation. Overall non-parametric diversity estimators ACE and Chao I and their confidence intervals were computed for a sample size of n = 3 ARMS.

When comparing pH sites within a locality, abundance per sampling unit was generally greater at higher versus lower pH sites [e.g. ARMS at Upa Upasina: control > medium and low; ARMS at Dobu: medium > low (Table 1)]. An exception was found at the control pH site at Dobu that displayed a very low abundance of invertebrates, only slightly higher than the low pH site at Dobu. At Dobu, the 56% drop in cryptofaunal abundance (individuals per ARMS) between medium and low pH sites (60.3 vs. 26.5 individuals per ARMS, Table 1) was statistically significant (ANOVA: F = 14.62, p < 0.001). However, at Upa Upasina the 34% drop in cryptofaunal abundance between control and medium or low pH sites (75.5 vs. 50.0 or 50.7 individuals per ARMS; Table 1) was not.

The abundance of crustaceans in dead coral heads from Upa Upasina also declined between the control and medium pH sites, with an average of 87 individuals per dead head at the control pH site and 51 individuals per dead head at the medium pH site (S1 Fig).

### 2. Overall diversity

The 1424 invertebrates from the ARMS clustered into 327 taxonomically distinct OTUs (213 in Upa Upasina and 181 in Dobu; Tables 1 and S2). Most OTUs were rare, either in terms of total numbers (166 or 50.8% were found only once) or spatial pattern of occurrence (in addition to singletons, 26 OTUs or 8.0% were only found in a single ARMS unit in abundance smaller than 5 individuals, and 27 OTUs were collected from multiple ARMS at just one pH site). Of the OTUs collected from more than one site, 67 or 20% were found at both localities, 34 or 10% were restricted to Upa Upasina, and 7 or 2% were only found in Dobu.

Only 22 of these OTUs (6.7%) had over ten individuals summed across all samples, 18 of which were present at both localities. The most abundant OTU, the shrimp *Palaemonella rotumana* (Borradaile, 1898), was found in high numbers (>15) at all sites except at the control pH site in Dobu, where only a single specimen was found (S2 Table).

**Fig 2:**
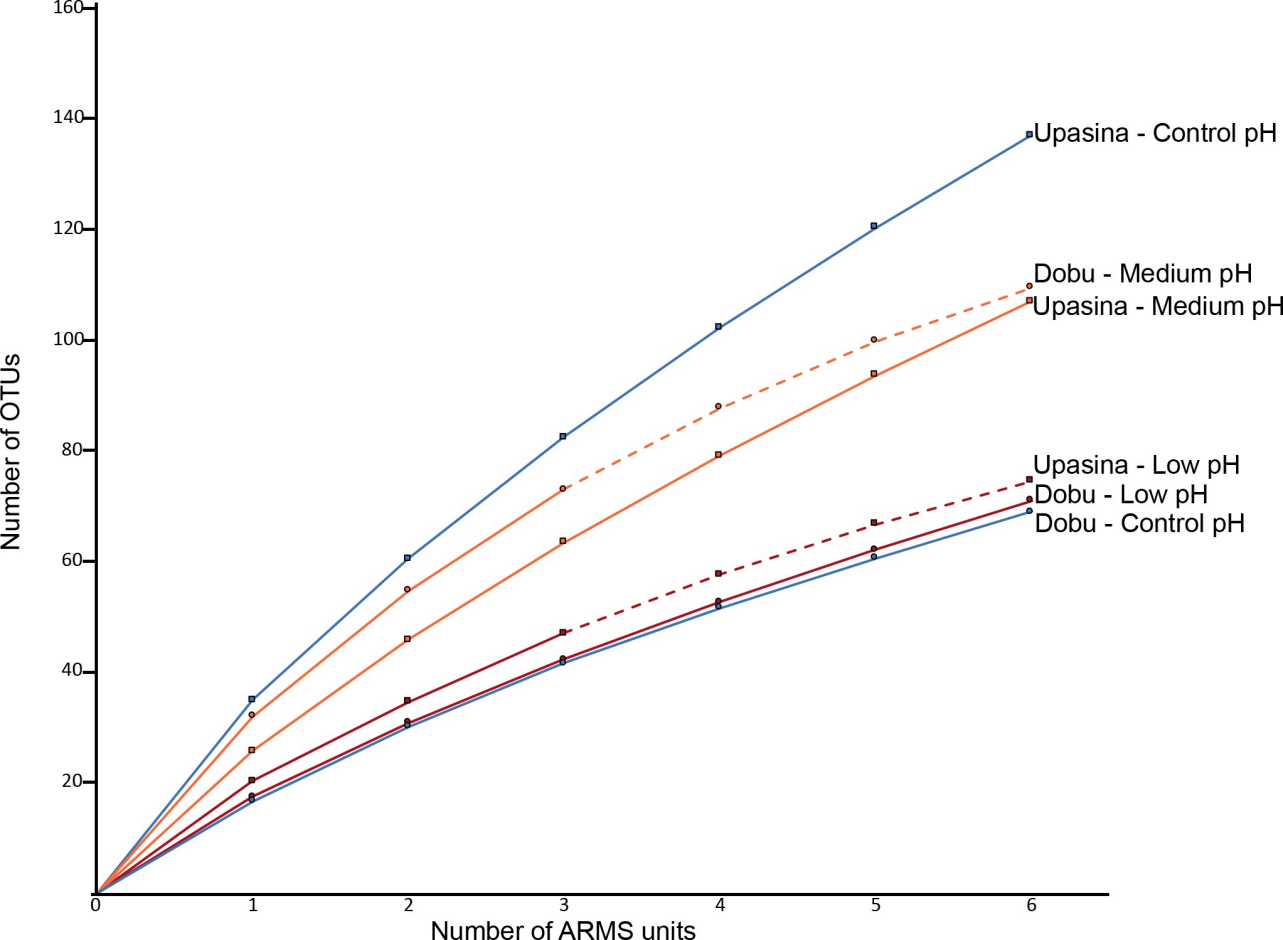
Sample rarefaction curves. Rarefaction curves for invertebrate assemblages of all ARMS units from Upa Upasina (square markers) and Dobu (round markers) in control (blue), medium (orange) and low (red) pH conditions. The dotted lines represent samples for which the sampling size was extrapolated to six ARMS units.

**Fig 3:**
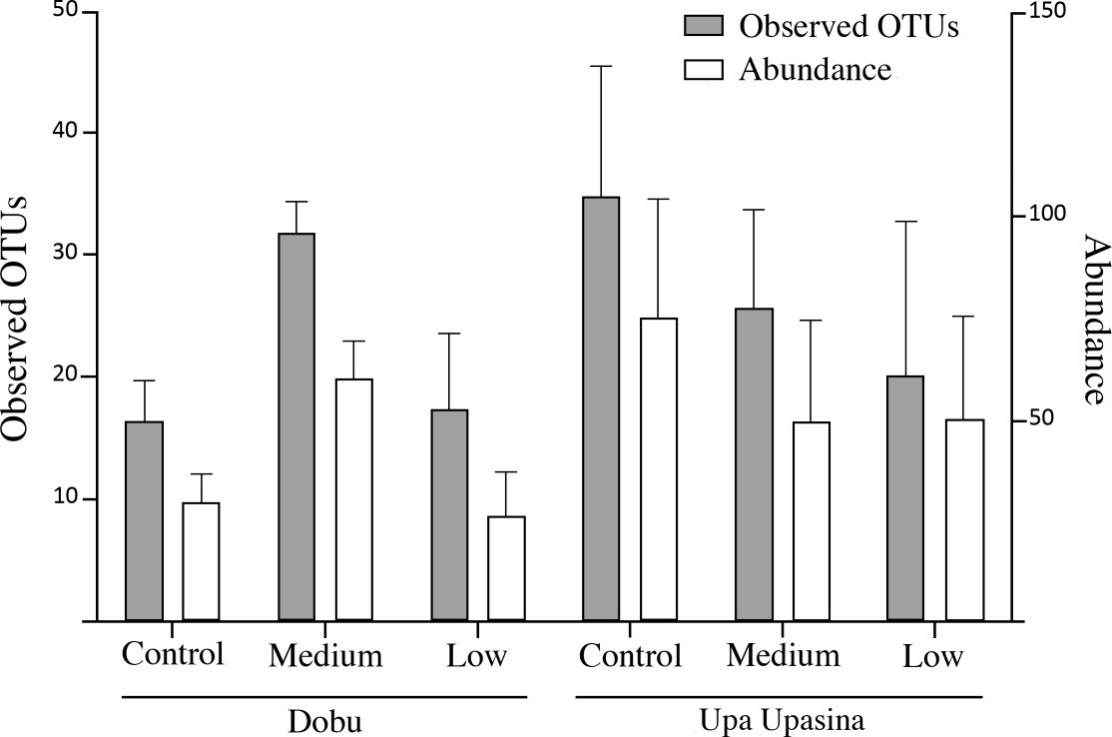
Mean abundance and diversity at each pH site. Shown are the number of taxa (observed OTUs) and individual abundances for each locality (Dobu and Upa Upasina) at the three pH conditions (control, medium and low pH). Error bars represent the standard deviation.

As was expected given the prevalence of rare species, none of the rarefaction curves for the six pH sites reached an asymptote (Fig 2), indicating that sampling was non-exhaustive (i.e., the number of individuals sampled was insufficient to accurately represent overall diversity). Diversity metrics (average number of OTUs per ARMS, Shannon diversity index, Chao and ACE diversity estimators) exhibited patterns similar to those observed with the abundance data. In general, higher pH sites tended to have higher diversity values within a locality (ARMS at Upa Upasina: control > medium > low; ARMS at Dobu: medium > low; Fig 3 and Table 1). However, like the abundance data, the control Dobu site had unusually low diversity (similar to the low pH site). We observed 53–57% drops in estimated diversity (ACE and ChaoI estimators) between the medium and low pH at Dobu, and 50–53% drops between the control and low pH at Upa Upasina. At Dobu, the decrease in the mean numbers of OTUs per ARMS (45% drop) and the Shannon index (from 3.21± 0.15 to 2.67 ± 0.35) between medium and low pH were statistically significant (ANOVA; F = 11.96, p = 0.001 and F = 5.72, p = 0.018, respectively). At Upa Upasina, the drop in Shannon diversity between control and low pH site (from 3.12 ± 0.15 to 2.48 ± 0.52) was also significant (ANOVA: F = 5.43, p = 0.021). However, the 42% drop in mean number of species per ARMS between the control and low pH site was not statistically significant.

As with abundances, the diversity of crustaceans in dead coral heads from Upa Upasina was higher at the control pH site (mean of 29 OTUs per dead head) than at the medium pH site (mean of 22 OTUs per dead head) (S1 Fig).

### 3. Patterns across phyla and families

At both localities, arthropods were the most abundant (74% and 55% of the individuals at Upa Upasina and Dobu, respectively), followed by mollusks (19% and 36% of the individuals at Upa Upasina and Dobu respectively). Arthropods represented >50% of the organisms at all sites except at the Dobu low pH site, where mollusks were the most abundant (55% of the total number of organisms). Other phyla (Echinodermata, Annelida and Sipuncula) represented a small portion of the total abundance (4.1%, 2.5% and 0.7% of organisms; both localities combined; Figs 5A and 5B and S2).

In contrast to the numerical dominance by arthropods at most sites, mollusks represented an important proportion of the diversity (OTUs). At Upa Upasina, arthropods represented 49% and mollusks represented 35% of the OTUs. At Dobu, in contrast, arthropods (35% of the OTUs) were less diverse than the mollusks (50% of the OTUs). As expected from their low abundances, Echinodermata, Annelida and Sipuncula also represented a small portion of the OTUs (7%, 6.1% and 1.8% respectively; both localities combined).

**Fig 4:**
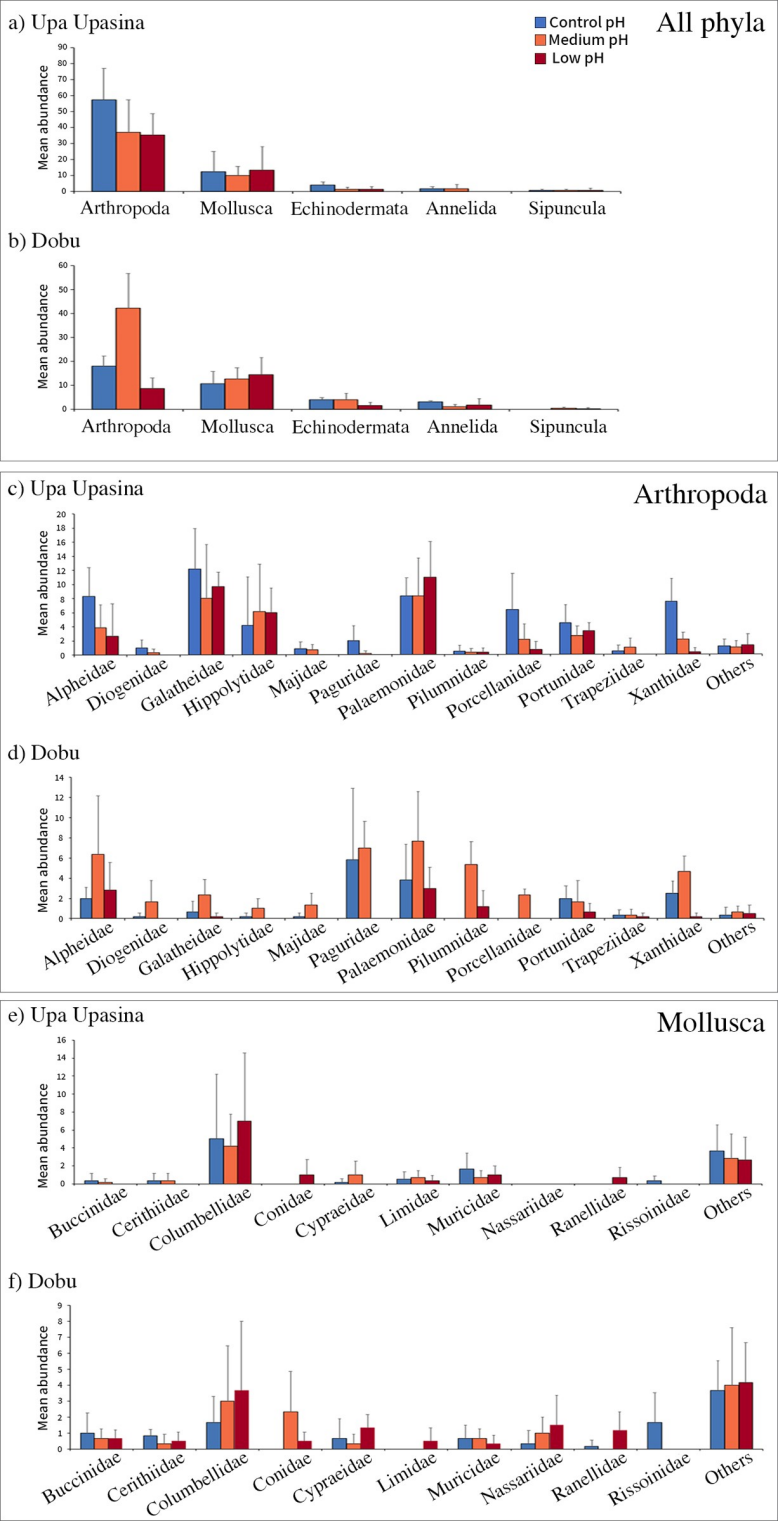
Average number of organisms per ARMS at each six pH sites at the phylum level and for the main arthropod and mollusk families. Mean abundance (with standard deviation) at the three pH conditions investigated [control (high), medium and low pH] at the phylum level at a) Upa Upasina and b) Dobu; for the main arthropod families at c) Upa Upasina and d) Dobu; and for the main mollusk families at e) Upa Upasina and f) Dobu.

**Fig 5:**
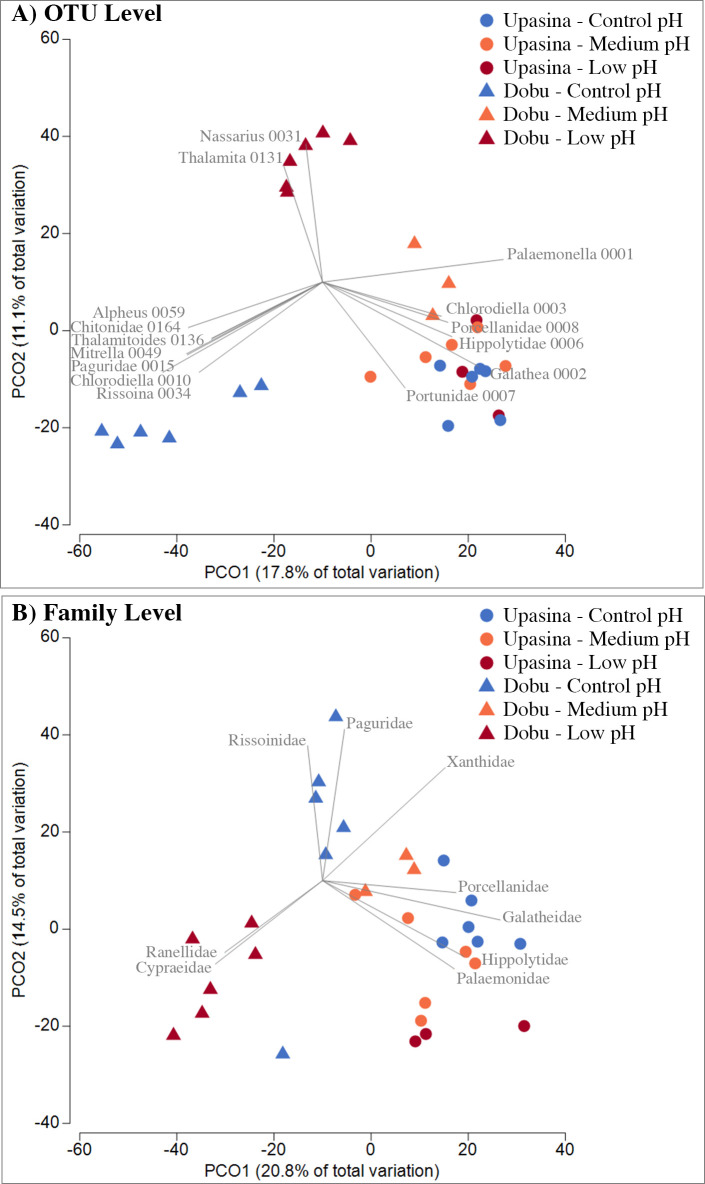
Principal coordinate analysis (PCoA) of invertebrate community composition in ARMS at the six pH sites. Principal Coordinate Analysis (PCoA) plots performed on Bray-Curtis dissimilarities of log-transformed abundances at the two localities (Dobu and Upa Upasina) and the three pH sites (low, medium and control pH) analyzed at A) the OTU level and B) the family level. Each point represents an ARMS sample. Pearson’s correlation vectors are overlaid on the PCoA plot (vector length greater than 0.6). OTUs are named by their lowest taxonomic rank followed by their OTU number.

Because of their numerical dominance, patterns in the overall invertebrate abundance (Fig 3) and arthropod abundance were similar at both Upa Upasina (Fig 4A) and Dobu (Fig 4B). Most arthropod families broadly followed the overall arthropod trend with some exceptions for the shrimps displaying a lightly calcified body (Hippolitidae and Palaemonidae) (Fig 5C and 5D). As with the broader scale patterns, the strongest differences among pH sites occurred in Dobu, whereas in Upa Upasina, the patterns were generally not statistically significant. For instance, arthropod abundance was significantly different between pH sites in Dobu (χ = 11.12, df = 2, p = 0.0038) but the observed decrease in arthropod abundance with declining pH in Upa Upasina was not statistically significant (p = 0.165). However, significant differences were observed in xanthid crabs abundance both at Upa Upasina (χ = 11.92, df = 2, p = 0.003), where the highest abundance was found at the control pH site, and at Dobu (χ = 16.21, df = 2, p = 0.016), where the highest abundance was found at the medium pH site.

Mollusks, in constrast, showed no trend towards lower abundance at low pH (Fig 5A and 5B). Within the mollusks, the abundant columbellids appeared to show slightly increased abundances in lower pH at both Upa Upasina (Fig 4E) and Dobu (Fig 4F) however this trend was not statistically significant.

For the Echinodermata, Polychaeta and Sipuncula, recorded overall abundances were too low to confidently assess differences between pH conditions. However, echinoderms (mainly represented by ophiuroids with 78% of sampled echinoderm individuals and 74% of echinoderm OTUs), showed a pattern of slightly higher abundances at higher pH sites at both localities (Fig 5A and 5B).

### 4. Community structure

The PCoA analysis revealed strong structuring for the ARMS invertebrate communities at Dobu, with samples clustering according to the three pH conditions when analyzed both at the OTU (Fig 5A) and family (Fig 5B) levels. At Upa Upasina, in contrast, there was much more overlap among the three pH sites. The 2-way PERMANOVA analysis revealed a significant effect of locality (F = 4.24, p < 0.001 at the OTU level; F = 5.00, p < 0.001 at the family level) and pH (F = 2.25, p < 0.001 at the OTU level; F = 2.10, p < 0.001 at the family level), as well as a significant interaction between pH and locality (F = 2.24, p < 0.001 at the OTU level; F = 1.82, p < 0.007 at the family level). The interaction was explored using one-way pairwise PERMANOVA analysis for individual locality datasets separately; this revealed significant differences among all three pH conditions for Dobu at the OTU and family level (p < 0.05), whereas at Upa Upasina only the two most extreme pH conditions were significantly different for both taxonomic levels (low pH vs control pH: p < 0.05).

SIMPER analysis (similarity percentages based on Bray-Curtis dissimilarity matrices) highlighted the families making the largest contributions to these patterns. For the decapods, families contributing the most to the differences were the alpheids and xanthids at both localities, the pagurids and the palaemonids specifically at Dobu, and the porcellanids and hippolytids at Upa Upasina. When looking at the abundance patterns, most of these groups showed a higher abundance at the higher pH conditions, except for the hippolytids and palaemonids, which were more abundant in lower pH conditions (Table 2 and Fig 5). For the mollusks, the differences for the two localities were mainly driven by the collumbellids, which were more abundant in the lower pH conditions.

**Table 2:** SIMPER analysis (similarity percentages based on Bray-Curtis dissimilarity matrices) showing the main contributors to the dissimilarities between pH conditions at both localities.

	Dobu		Upa Upasina	
Control vs Low pH	Overall dissimilarity	69.35	Overall dissimilarity	54.04
	Paguridae	6.58	Xanthidae	7.94
	Xanthidae	5.90	Alpheidae	7.48
	Columbellidae	4.86	Porcellanidae	6.09
	Alpheidae	4.33	Columbellidae	5.95
	Palaemonidae	4.28	Hippolytidae	5.32
Control vs Medium pH	Overall dissimilarity	62.86	Overall dissimilarity	50.92
	Pilumnidae	5.65	Hippolytidae	5.82
	Paguridae	5.24	Porcellanidae	5.26
	Ophiocomidae	4.99	Columbellidae	5.06
	Porcellanidae	4.95	Alpheidae	4.37
	Alpheidae	4.34	Galatheidae	4.10
Medium vs Low pH	Overall dissimilarity	69.59	Overall dissimilarity	52.49
	Paguridae	6.48	Alpheidae	6.56
	Xanthidae	6.18	Columbellidae	6.51
	Pilumnidae	5.23	Hippolytidae	6.21
	Porcellanidae	4.57	Xanthidae	4.92
	Galatheidae	4.02	Porcellanidae	4.59

For each pairwise comparison, the overall dissimilarity as well as the top five families contributing the greatest percentage of overall dissimilarity between pH conditions are shown for each locality.

## Discussion

### Using ARMS to characterize the effects of ocean acidification on the coral-associated cryptofauna

Since the initial use of ARMS as a sampling method for structurally complex benthic habitats [28,34], they have been deployed worldwide to investigate and monitor marine biodiversity [28,30,31] or detect the presence of invasive or non-indigenous species [47]. ARMS have also been used to characterize biogeographical patterns [33,48] and the impacts of altered environments on benthic communities [49] and represent an efficient standardized tool with which to monitor the effects of environmental variables on marine creatures [50]. This study represents the first attempt to use ARMS to quantify the effects of ocean acidification on the cryptofauna.

One obvious limitation of using artificial sampling structures is their ability to properly represent the surrounding communities in the limited amount of deployment time. The effectiveness of using artificial structures to study natural communities was evaluated by Plaisance *et al*. [29]; they found that ARMS deployed for a one-year period in Australia on the Great Barrier Reef (GBR) displayed slightly lower species richness and abundance but were able to capture similar diversity patterns over a longitudinal gradient when compared with natural substrata (*i*.*e*. dead *Pocillopora* sp. coral colonies). During the present study, we conducted a similar comparison in Upa Upasina on the crustacean diversity found on dead *Pocillopora* sp. colonies and ARMS in high and medium pH conditions. Similarly to the GBR [29], the diversity and abundance of crustaceans on ARMS were 66%-76% of that observed on dead coral heads, the difference was driven particularly by groups that are highly adapted to specific coral structures (e.g. Xanthidae and Alpheidae), but overall patterns across the pH gradient were similar (S1 Fig). Thus quantitative sampling from standardized structures can be used for diversity comparisons between sites, and are particularly helpful when comparing locations with different levels of benthic structural complexity. Another advantage of using ARMS is that results can be interpreted in the context of a wider monitoring efforts (*e*.*g*. the Global ARMS Program (https://oceanarms.org/)), thereby permitting geographic, temporal, and environmental comparisons.

### Differences among pH levels in cryptofauna abundance, diversity, and taxonomic composition

Our study of the cryptofauna associated with naturally acidified coral reefs confirms the findings of previous research investigating the response of invertebrates in other acidified habitats. Our analysis showed reduced overall diversity and abundance of coral reef invertebrates in lower pH seawater. Community composition was also significantly different among all three pH sites at Dobu and between the two most extreme pH conditions (low and control) at Upa Upasina revealing deep community shifts in acidified environments.

These patterns broadly agree with previous observations of other invertebrates from this region that used different sampling methods and slightly different sampling sites. The cryptic invertebrates from this study displayed somewhat stronger declines in diversity at Upa Upasina (mean numbers of OTUs per ARMS at the low pH site of 58% of the control site) than the study by Fabricius et al. [12] based on larger free-living invertebrates (> 1cm in size) which showed a diversity in low pH conditions of 77% of the control pH conditions. This difference could be a result of the different taxonomic resolution used in the two studies. In the present study, DNA-assisted taxonomy allowed for a more precise resolution when compared with visual identifications during transect surveys; differences between DNA-based and visual surveys are likely to be particularly marked in highly biodiverse settings where a small proportion of the diversity has been characterized, as is the case for the reefs of Papua New Guinea.

In contrast, Fabricius et al. [12] reported slightly larger differences in invertebrate densities, with values at low pH conditions of 43% of those observed in control pH conditions (ARMS cryptofauna abundances in low pH condition were 66% of those observed at the control site). This might be due in part to the fact that the ARMS might have locally increased the availability of three-dimensional space for invertebrates, thereby offsetting some of the effects associated with loss of structurally complex corals. However, it is also possible that the availability of hiding places for very small mobile invertebrates may be less impacted by acidification than they are for larger organisms. In the case of the cryptofauna, several studies have shown that in present-day pH environments, coral reefs exhibiting a certain degree of degradation can hold a higher biomass/density and species richness of cryptofaunal organisms when compared to live coral habitats [51]. The presence of additional coral rubble in impacted sites could therefore have provided more shelter space for these smaller species, whereas larger invertebrate species might face higher predation pressure in the more open, less structurally complex habitats that characterize lower pH conditions. At the lowest pH values found closest to the seeps, however, even coral rubble becomes scarce.

Research on other acidified systems have also reported major compositional shifts in benthic invertebrate communities in low pH conditions, with taxon-specific effects [52]. In our study, the two most abundant and diverse phyla found in the ARMS were arthopods and mollusks. Most crustacean families were negatively affected by lower pH conditions, as was also found by Fabricius et al. [12]. This pattern runs contrary to the findings of laboratory physiological studies, which typically show crustaceans to be less sensitive than mollusks, with their calcification process appearing to be less vulnerable to low pH conditions [11,53,54]. The greater sensitivity of crustaceans to ocean acidification points to the importance of indirect negative effects such as enhanced predation on species living on structurally simplified reefs. Similarly, Smith et al. [25] reported that the abundance of copepods in the demersal zooplankton at low pH sites was up to 71% lower compared to the control sites. On the other hand, Allen et al. [26] found mixed results for various groups of microcrustaceans recruiting into scouring pads, with taneids being significantly more abundant at elevated CO_2_ sites while copepods and amphipods showed strong declines in higher CO_2_ concentrations and lower pH environments. Decapods, which constitute the bulk of the diversity found in the >2 mm fraction of the ARMS, were not abundant in their study and did not display a clear pattern relative to the pH. The pattern of decreased abundance and diversity of decapods in lower pH conditions is consistent with findings in temperate acidified systems in the Mediterranean sea where decapods were entirely absent in extreme pH conditions whereas microcrustaceans thrived [55,56].

In contrast to arthropods, mollusks were more abundant in lower pH conditions at both sites in our study. This is somewhat surprising, as Allen et al. [26] found high sensitivity of juvenile gastropod and bivalve recruitment to lower pH corresponding to the known sensitivity of their larval stages to acidification [54]. Juvenile gastropods in temperate systems have also been shown to be sensitive to lower pH consitions [52]. However, our study targeted groups that are mostly in their adult life stage when collected, as did Fabricius et al. [12], where no significant change but a similar pattern of greater density of mollusks at lower pH sites was observed when compared with control pH sites. Other temperate studies have also reported adult gastropod tolerance to decreasing pH up to a threshold, beyond which they are entirely absent [55,56], but that pH threshold was not reached at our study sites. These observations may reflect positive indirect effects of ocean acidification over a pH range where organisms are still able to physiologically counteract the consequences of lowered pH. For example, an increase in CO_2_ concentrations acts as a direct resource for photosynthetic organisms, thereby producing an increased supply of food in the form of algal biomass and benefiting herbivorous species such as many gastropods [57,58] and other herbivorous taxa such as sea urchins [24]. In our study, the trend of increased abundance at lower pH was mainly driven by the family Columbellidae, the most abundant family in our samples; all three molluscan species with overall abundances higher than 15 individuals, *Pardalinops marmorata* (Gray, 1839), *Mitrella moleculina* (Duclos, 1840) and *Euplica turturina* (Lamarck, 1822) are members of this family. Unfortunately, the feeding types of the very diverse columbellids are extremely variable, including grazers, scavengers and detritivores [59], making it very difficult to link our observations to scarce information on the feeding ecology of these species. The apparent tolerance of low pH environments by this heterogenous group of mollusk species shows that the response to ocean acidification is complex and species-specific. Data on life history traits of cryptic coral reef species are largely lacking in the literature, and the ecological mechanisms explaining these observations are yet to be resolved.

### Differences between localities

Despite their relative proximity to each other, the comparison of diversity and community composition patterns at the two localities investigated in this study revealed important differences. For example, some comparisons between the two localities of sites with similar pH conditions (e.g. low pH Upa Upasina versus low pH Dobu, medium pH Upa Upasina versus medium pH Dobu) revealed similar diversity and abundance patterns (Table 1 and Figs 3 and 4), but the community composition was highly distinct (Fig 5).

Most notably, the control site in Dobu displayed an unusually low number of both individuals and OTUs, as well as marked differences in community composition patterns that were consistent across the six replicate ARMS samples. These low numbers are unusual for ARMS deployed in coral reef systems, and are unexpected because of the location of this study in the mega-diverse Coral Reef Triangle [60]. For example, studies using similar sampling and analytical methods on Red Sea reefs of Jordan [31] and Saudi Arabia [33], regions that are less diverse, reported 20.0–27.6 OTUs per ARMS compared to 16.7 OTUs per ARMS in the Dobu control site, and the Jordan study reported over twice the abundance of individuals per ARMS than was observed at the Dobu control site (66.2 versus 30). Indeed, the average value of 16.7 OTUs per ARMS observed at the Dobu control site is comparable to diversity results reported from ARMS deployed subtidally on oyster reefs in subtropical Florida (16 OTUs/ARMS; [30]), a much less diverse habitat than tropical coral reefs. Similarly, when examining the diversity of crustaceans only, Plaisance et al. [29] found an average of 22 crustacean OTUs on ARMS deployed on forereef habitats in Heron Island, Australia (Great Barrier Reef) and 18 OTUs on fringing reefs in French Frigate Shoals (Northwestern Hawaiian Islands), with average abundances of more than 50 crustaceans per ARMS [28], whereas the comparable mean figures at the Dobu control site for crustaceans were 7.3 (± 2.9) OTUs and 18 (± 4.3) individuals per ARMS.

This suggests that the Dobu “control” location was locally depauperate for cryptofauna, for reasons that are undetermined. Other studies have also noted biological differences between Upa Upasina and Dobu. For example, Kenkel et al. [61], when examining global gene expression patterns in corals and their intracellular algal symbionts, detected bigger differences between the reefs than between pH levels. Comparing the two control sites, Russell et al. [62] reported lower seagrass biomass at Upa Upasina than Dobu, and Uthicke et al. [63] reported differences in the communities of foraminifera. Allen et al. [26] also found differences between the two locations, including much higher levels of decapod recruitment at Upa Upasina than at Dobu.

The differences in patterns across the pH gradient for the two localities may reflect the fact that the control and vent sites at Upa Upasina were topographically similar and located on a continuous strip of reef separated only by 500 m, whereas the control site for Dobu was on a different patch reef 2.5 km from the lower pH sites slightly further away from the shore on a more wave and current protected side of the island [21]. The fact that the control site of Dobu had lower diversity and abundance of invertebrates than even the low pH site of Upa Upasina highlights the complexity of characterising the diversity of strongly heterogeneous habitats like coral reefs and the difficulty of untangling the effect of one particular driver like seawater pH from other drivers of diversity. The complexity of responses observed in our study, in particular the differences between localities, indicated that the effects of localized reduction in pH are imbedded in other ecological responses, which may be of comparable or greater strength.

Another limitation of *in situ* natural experiments is the greater variability in seawater chemistry observed at CO_2_ seeps [12] that exceeds the oscillation in most coastal waters. The fluctuations measured do not always correspond to the natural diel periodic changes driven by community photosynthesis and respiration and may interfere with our ability to precisely quantify the impacts of OA on marine species [64].

Nevertheless, our data suggest that the progressive decline in pH due to ocean acidification, superimposed over other natural environmental gradients, can greatly reduce the number and diversity of reef cryptofauna and induce important shifts in the composition of reef-dwelling invertebrate communities, with potentially severe consequences for many reef functions.

## Supporting information

S1 Fig Comparison of the diversity and abundance of crustaceans in ARMS and natural substrate. Bar plot depicting the average number of OTUs (grey) and individuals (white) found in sampling units for each pH condition (control and medium pH) / sample type (ARMS and coral heads) investigated. Error bars represent ± 1SE.
(TIF)

S2 Fig Composition bar plot. Bar plot depicting the relative abundance of the five phyla found in the ARMS at Dobu and Upa Upasina for the three pH conditions investigated.
(TIF)

S1 Table Seawater carbonate chemistry. Average water temperature and carbonate parameters at the control, medium, and low pH sites at Dobu and Upa Upasina.
(DOCX)

S2 Table OTU table. Table containing the taxonomic identification and number of sequences that were observed for each Operational Taxonomic Unit (OTU) in each ARMS sample for the two localities (Upa Upasina and Dobu) and three pH conditions investigated. The name of the ARMS sample is given as: Locality_ARMS_#_pH. The pH categories are: C = control, M = medium and L = low).
(XLSX)
