# Supplementary figures and images for: Effects of low pH on the coral reef cryptic invertebrate communities near CO2 vents in Papua New Guinea
Source: PLoS One. 2021 Dec 15;16(12):e0258725. doi: 10.1371/journal.pone.0258725 (PMC8673656; doi:10.1371/journal.pone.0258725)

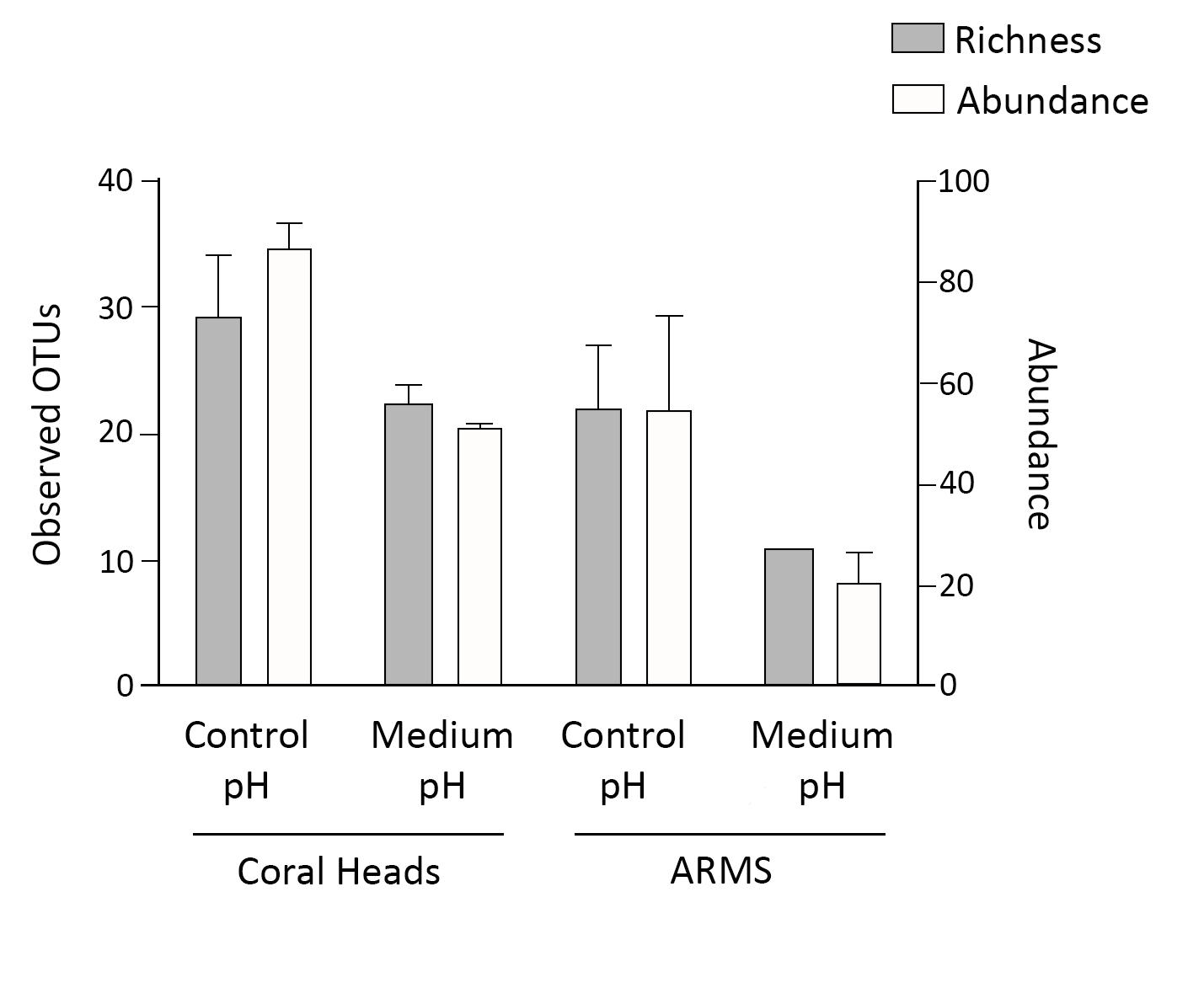

Supplement: S1 Fig — Bar plot depicting the average number of OTUs (grey) and individuals (white) found in sampling units for each pH condition (control and medium pH) / sample type (ARMS and coral heads) investigated. Error bars represent ± 1SE. (TIF) [file pone.0258725.s001.tif]

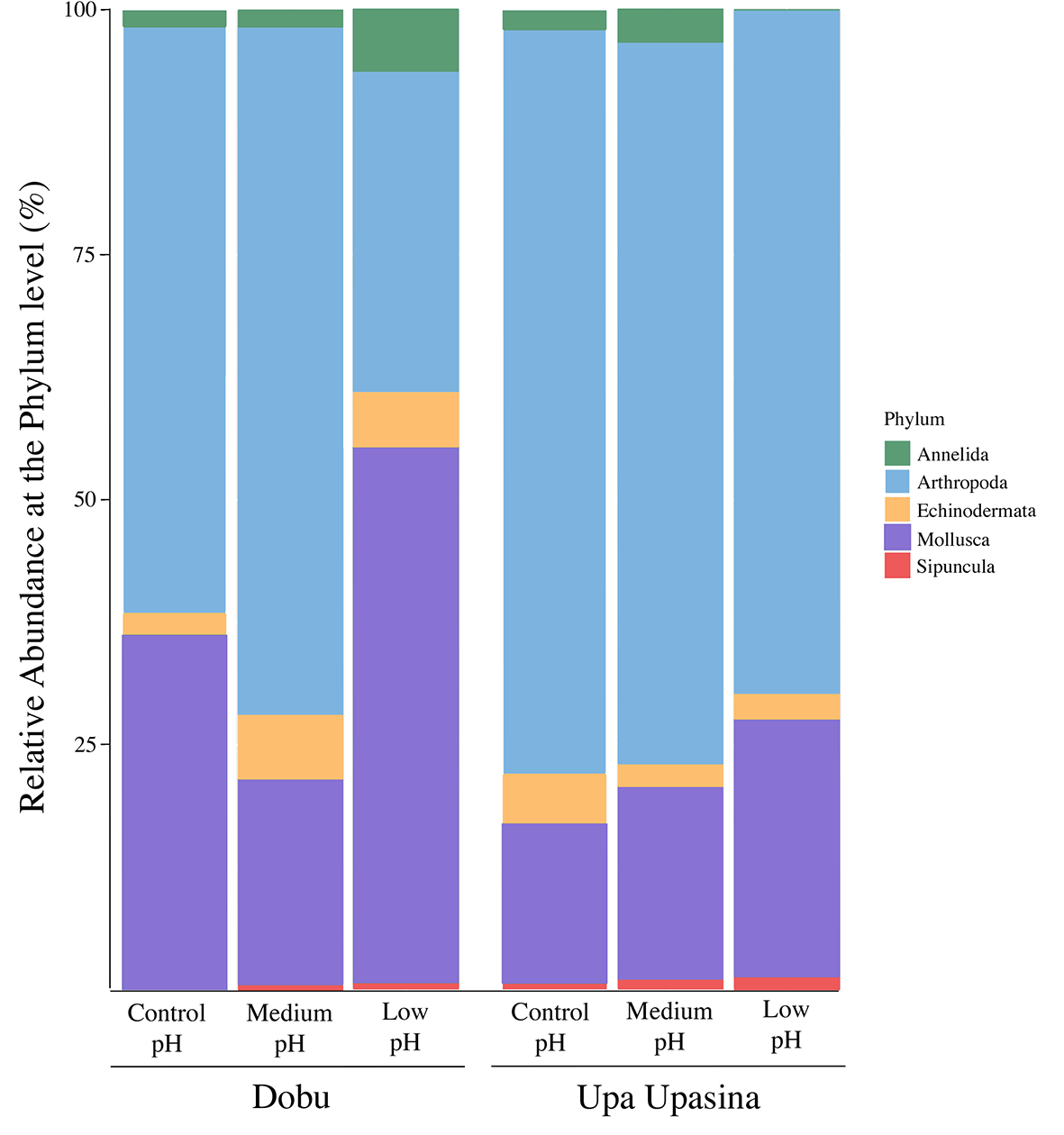

Supplement: S2 Fig — Bar plot depicting the relative abundance of the five phyla found in the ARMS at Dobu and Upa Upasina for the three pH conditions investigated. (TIF) [file pone.0258725.s002.tif]
