# Supplementary material for: Effects of low pH on the coral reef cryptic invertebrate communities near CO2 vents in Papua New Guinea
Source: PLoS One. 2021 Dec 15;16(12):e0258725. doi: 10.1371/journal.pone.0258725 (PMC8673656; doi:10.1371/journal.pone.0258725)
Supplement: S1 Table — Average water temperature and carbonate parameters at the control, medium, and low pH sites at Dobu and Upa Upasina. (DOCX) [file pone.0258725.s003.docx]

**S1 Table. Seawater carbonate chemistry.** Average seawater temperature and carbonate parameters at the control, medium, and low pH sites at Dobu and Upa Upasina.

| Locality | pH Site | Temperature (°C) | 05^th^-95^th^ | pH | 05^th^-95^th^ | TA (mmol kg^-1^) | DIC (mmol kg^-1^) | pCO_2_ (µatm) | Ωar |
| --- | --- | --- | --- | --- | --- | --- | --- | --- | --- |
| Dobu | Control | 29.70 (0.009) | 29.7-30.7 | 7.99 | 7.88 - 8.11 | 2221 (2.75) | 1948 (0.90) | 471 (2.90) | 3.17 (0.04) |
|  | Medium | 28.68 (0.006) | 28.6-29.3 | 7.85 | 7.39 - 8.11 | 2255 (1.76) | 2034 (7.22) | 630 (24.14) | 2.66 (0.07) |
|  | Low | 29.63 (0.010) | 29.9-30.7 | 7.64 | 6.99 - 7.97 | 2270 (7.26) | 2182 (31.66) | 1609 (305.02) | 1.47 (0.22) |
| Illi | Control | 29.66 (0.006) | 29.5-30.7 | 8.01 | 7.86 - 8.17 | 2223 (1.50) | 1952 (10.95) | 515 (25.25) | 3.17 (0.10) |
|  | Medium | 29.50 (0.009) | 29.6-30.5 | 7.85 | 7.59 - 8.02 | 2295 (16.52) | 2003 (19.69) | 490 (40.73) | 3.32 (0.16) |
|  | Low | 29.44 (0.012) | 29.7-30.7 | 7.75 | 7.26 - 8.00 | 2267 (14.21) | 2046 (19.05) | 708 (60.36) | 2.64 (0.13) |

Temperature and pH (presented at the total pH scale) values with 5^th^ and 95^th^ percentiles were measured using Sea-bird Scientific and SeaFET sensors deployed *in situ* during three expeditions between 2012-214. Measurements were recorded every 10 minutes for 24 hour periods. Total alkalinity (TA) and dissolved inorganic carbon (DIC) were measured from discrete seawater samples (N = 2-14). The other carbonate system parameters, pCO_2_ and aragonite saturation state (Ωar) were calculated from measured values of temperature, TA and DIC. Standard errors are shown in parentheses.
